# Live imaging of bacterial actin MreBs from *Spiroplasma* causing helicity switching of a minimal synthetic cell

**DOI:** 10.2142/biophysico.bppb-v23.0017

**Published:** 2026-05-13

**Authors:** Yoshiki Tanaka, Hana Kiyama, Yusuke V. Morimoto, Takayuki Nishizaka, Makoto Miyata

**Affiliations:** 1 Graduate School of Science, Osaka Metropolitan University, Osaka 558-8585, Japan; 2 Department of Physics, Gakushuin University, Tokyo 171-8588, Japan; 3 The OMU Advanced Research Center for Natural Science and Technology, Osaka Metropolitan University, Osaka 558-8585, Japan; 4 Department of Physics and Information Technology, Kyushu Institute of Technology, Fukuoka 820-8502, Japan

**Keywords:** JCVI-syn3B, *Mycoplasma*, FRAP, Helix, Synthetic biology

## Abstract

*Spiroplasma* swim by switching the handedness of their helical bodies between right- and left-handed. Helicity formation and switching can be reconstituted in an immotile minimal synthetic bacterium, JCVI-syn3B by introducing a pair of bacterial actins, MreB4 and MreB5 from *Spiroplasma eriocheiris*. However, the mechanism is unknown. Here, to elucidate this mechanism, we analyzed MreB behaviors optically. We tried MreB4 fluorescence labeling by protein fusion. Labeling was unsuccessful because the fusion of fluorescent proteins or peptides at 16 different positions resulted in immotile cells. These results may suggest that MreB4 has many interfaces interacting with other proteins. To obtain suggestions for roles of MreB4 and MreB5, we tried induction of individual MreBs. Induced expression of MreB4 in cells with constitutive MreB5 expression resulted in earlier onset and higher frequency of motile cells, distinct from the results of constitutive MreB4 and inducible MreB5. Next, the behavior of labeled MreB5 was analyzed by photobleaching and photoactivation, suggesting static behavior of MreB5 during cell movements. Cell treatment with A22, an MreB polymerization inhibitor caused helix deformation, movement stall, and diffusion of MreB5 fluorescence, suggesting that A22 sensitive MreB5 interaction should be involved in helix formation and motility. These results suggest that the movement is caused by conformational change of MreB5 filament induced by MreB4 without obvious replacements of MreB5 subunits.

## Significance

*Spiroplasma* MreB4 and MreB5 are unique bacterial actins that cause motility with helicity switching, a different mechanism from other actins. Here, we investigated the helicity switching reconstituted in a minimal synthetic bacterium. Fluorescently traced MreB5 behaviors suggested that the movement is caused by conformational change of MreB5 filament induced by MreB4 without obvious replacements of MreB5 subunits.

## Introduction

**Figure 1:**
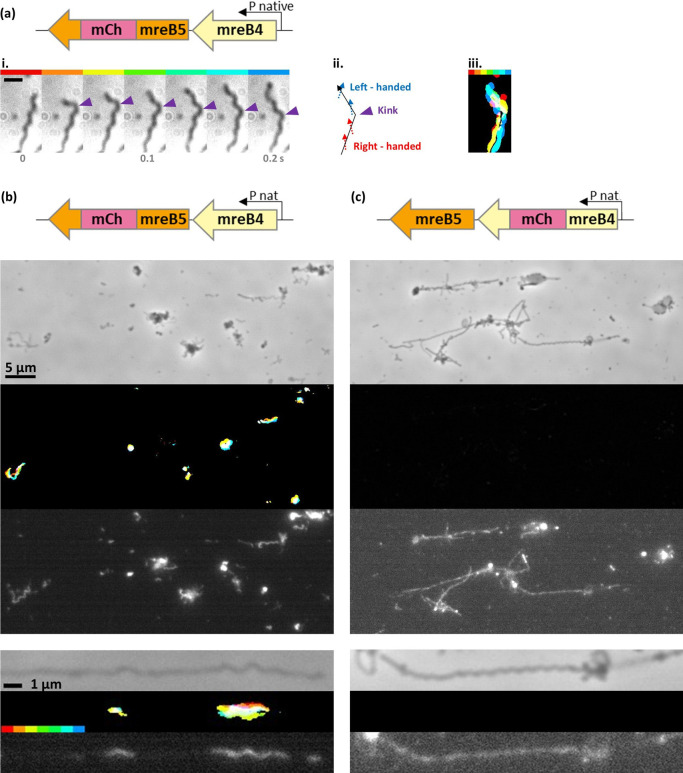
Cellular localization of each MreB fused with mCherry. Gene constructs are shown on the top of each panel. (a) Helicity switching manner of syn3 expressing MreB4 and MreB5. (i) Video frames of syn3 cell switching helicity handedness. The switching point (kink) is marked by a purple triangle. (ii) Schematic cell characterization for the last frame of (i). Red and blue dotted arrows show the cell helicity. (iii) Rainbow trace, where each slice in (i) was colored from purple to red and integrated. (b) The strain expressing MreB4 and MreB5m. (c) The strain expressing MreB4m and MreB5. (b, c) Upper field images and lower cell images are shown for phase contrast, rainbow trace, fluorescence, from the top. In the rainbow trace, non-moving parts turned black. Excitation light was 10 times more intense for MreB4 than that for MreB5.

*Spiroplasma eriocheiris*, a crustacean parasite [1], belongs to the wall-less bacterial class Mollicutes [2,3]. It swims by switching its helical handedness between right and left. The boundary of the handedness called “kink” propagates along the axis, rotating the cell body to push water backward. They have no structural similarities with the machineries of Spirochetes helix rotation [4–6]. The *Spiroplasma* motility machinery is driven by a ribbon-like structure positioned along the innermost line of the helix [5,7]. The structure is composed of the *Spiroplasma*-specific protein Fibril, and five actin homologs MreB1-5 [7–11]. Previously, our group transferred genes of these proteins to a minimal synthetic bacterium JCVI-syn3B (syn3B) [12,13], which possesses a minimal set of genes for reproduction. The characteristic of minimal gene set can eliminate the possibility that other *Spiroplasma*-specific proteins are involved in the motility. Helicity switching was reconstituted by specific MreB pairs, MreB5–4, 5–1, 2–4, and 2–1 (Fig. 1a) [14]. MreB is a bacterial actin homolog generally acting as a scaffold for cell wall synthesis and is not related to motility in most bacteria [15]. MreB5 labeling was successful via fusion with mCherry immediately after Y218 out of the total 350 residues, while retaining motility. The fluorescence was observed through the entire cell length [14].

Focusing on eukaryotic actins, they drive motility by two mechanisms [16]. One is contraction as actomyosin, and the other is filament elongation [17]. *Spiroplasma* motility is distinct from them because it is caused by only two MreB isoforms without actin regulatory proteins. This force-generating mechanism may be a hint to understand the divergency and evolution of molecular motors. Moreover, the mechanism may be applicable to actuators of “nanorobot” based on artificial liposomes.

In this study, we manipulated the expression level and fluorescence labeling to outline the roles of MreB4 and 5. Then, we examined MreB5 dynamics in the cell movements by using photobleaching and photoactivation analyses and a polymerization inhibitor, A22 [18].

## Materials and methods

### Bacterial strains and culture conditions

*S. eriocheiris* (TDA-040725-5T) and JCVI-syn3B (GenBank, CP069345.1) were cultured in SP4 medium [19] at 30°C and 37°C, respectively, until they reached an optical density of 0.02–0.03 at 620 nm. For DNA manipulation, *Escherichia coli* strains DH5α and iVEC3 were used.

### Gene manipulation of syn3B

DNA manipulation and JCVI-syn3B transformation were performed as previously described [20–22]. The plasmids and primers used in this study are shown in Supplementary Figure S1 and listed in Table S1, respectively. pSeN540 and pSeN540mC5 were prepared from pSeW545 and pSeW545-F5 [14], respectively by replacing the Ptuf promoter with the native promoter of MreB4. The tetracycline-inducible module was introduced from pSD079. A codon optimized PAmCherry gene was synthesized by the GenTitan Gene Fragment service (GenScript, NJ, US). For DNA assembly In-Fusion® HD Cloning Kit (Takara Bio Inc., Kusatsu, Japan), NEBuilder® HiFi DNA Assembly Master Mix (New England Biolabs, Inc., MA, USA) [22], and *Escherichia coli* (iVEC3) were used (Supplementary Table S1).

### Cell treatments and optical microscopy

The following methods were used unless otherwise described. The cultured cells were centrifuged at 8,000×g for 5 min and suspended with supernatant at 5-fold cell density, if the cell density is not enough. The cell suspension was mixed in a ratio of 3 to 2 with a solution consisting of 20 mM 4-(2-hydroxyethyl)-1-piperazineethanesulfonic acid (HEPES), 330 mM NaCl, 0.8% Methylcellulose, and flowed into the tunnel chamber [23] assembled with coverslips cleaned by KOH saturated ethanol. After 5–10 min, floating cells were removed by a flow of 30 μl solution consisting of 0.4% Methylcellulose, 20 mM HEPES, 330 mM NaCl. A22 (Cayman Chemical Company, Michigan, US) was dissolved in DMSO to be 10 mM. For A22 treatment, the liquid in a tunnel was replaced with a solution consisting of 250 μM A22, 20 mM HEPES, 330 mM NaCl, 0.4% Methylcellulose. Cells were observed by an inverted microscope IX71 (Evident, Tokyo, Japan) with a UPlanSApo 100×/1.4 NA Ph3 and Complementary metal oxide semiconductor (CMOS) camera (DMK33UX174, The Imaging Source, Bremen, Germany). Images were captured at 30 frames per second (fps) and analyzed by ImageJ 1.54g.

For the identification of moving parts, the darkest value of each pixel among the movie frames (Image>Stack>Z projection) was subtracted from each frame to turn non-moving parts black. Then, the cell images with 1/30 s interval were binarized, colored from red to blue, and integrated to be “rainbow trace”. Cells including moving parts were judged as “moving cell”. To evaluate cell behaviors linked to promoter, the cultured cells were centrifuged at 9,000×g for 8 min and suspended with a phosphate-buffered saline (PBS) composed of 75 mM sodium phosphate (pH 7.3), 68 mM NaCl, 20 mM sucrose, at 20-fold cell density for observation.

### SDS-PAGE for protein expression analysis

For the expression induction, tetracycline dissolved in ethanol at 4 mg/ml was added to the culture at a final concentration of 0.4 μg/ml. The culture was then incubated at 37°C for various times, centrifuged at 13,200×g for 5 min, repeated twice and resuspended in 1×PBS at 50-fold cell density, lysed with 1/3 volume of SDS buffer (10% SDS, 20% Glycerol, 0.25M Tris-HCl pH6.8, 0.1% Bromophenol Blue, 20% β-mercaptoethanol) and ultrasonic treatment. The lysate was heated at 95°C for 3 min and subjected to SDS-10% or 12.5% PAGE. Loading volumes were normalized by culture ODs.

### Fluorescence microscopy, photobleaching and photoactivation

For fluorescence recovery after photobleaching (FRAP), cells were observed by an inverted microscope IX83 with a UPlanSApo 100×/1.45 NA Ph3 (Evident) and an Electron multiplying charge-coupled device (EMCCD) camera (ixon897, Andor, Belfast, UK), and a fluorescence filter set (Ex. 530–550 nm, Dichroic 570 nm, Em. 604–644 nm). Images were captured at 40 fps. Fluorescence imaging was done with 10% of the maximum intensity of LED light source (~2 W at the sample plane without a glass; U-LGPS, Evident). For bleaching, the LED light was manually switched to the maximum intensity and irradiated as a circular area with 10 μm diameter. Timelapse images were captured at 1 fps with 1/30 s exposure. For chemical fixation, cell culture was mixed in a ratio of 1 to 1 with an 8% paraformaldehyde solution, incubated at 22–27°C for 10 min, and rinsed with 1 M glycine. Fixed cells were centrifuged at 12,000×g for 10 min at 10°C and suspended with a solution consisting of 20 mM HEPES, 330 mM NaCl to be 2-fold cell density of the culture. Due to the small size of JCVI-syn3B cells, a circular bleached area was impossible unlike common way in FRAP. Additionally, tracking fluorescence intensity at a specific region was challenging due to cell movements. Therefore, we focused on extended helical cells sticking to glass. A part of extended cell bound to the glass was bleached, and pixel values were profiled perpendicular to the cell axis, with width of 5 pixels. The fluorescence intensity of a frame was quantified by subtracting the minimum value from the maximum value in the selected regions.

For photoactivation, we irradiated 352–402 nm light, by a pattern illuminator (LEOPARD2-White, OPTO-LINE Saitama, Japan). In this setup, cells were observed by IX73 equipped with a Scientific complementary metal oxide semiconductor (sCMOS) camera (Zyla4.2, Andor, UK). PAmCherry signals were detected by using a filter set (IX3-FMCHEXL, Evident; Ex.565–585 nm, Dichroic.595 nm, Em.600–690 nm).

## Results and discussion

### New constructs for adequate protein expression

To improve MreB expression levels, we replaced the Ptuf and Pspi promoters by the native promoter of MreB4 (Pnat) derived from *S. eriocheiris* (Supplementary Fig. S1). In these strains, the expression of MreB4 and 5 was 2.6±0.4- and 9.3±1.1-fold higher than those in the previous strain, respectively (Supplementary Fig. S2). The ratio of moving cells increased from 17.5% to 50.4% (Supplementary Movie S1). Previous strains expressing only MreB4 or MreB5 under the Pspi promoter did not show helical shape or movements [14]. However, new strains under Pnat promoter resulted in non-moving but helical cells with some ratio (Supplementary Movie S1). The protein amounts estimated from band intensity were 43±7 and 14±3-fold higher than the previous strains for only MreB4 or MreB5, respectively (Supplementary Fig. S2).

### Localization of fluorescently labeled MreBs

To examine the roles of MreB5 in elongation and motility of a cell, we observed the localization of mCherry-labeled MreB5 (MreB5m) in various forms of cells. Typically moving cells showed kink traveling along the cell body (Fig. 1a, Supplementary Movie S2). Fluorescence signals were observed in almost all the cells, localizing at moving and helical parts (Fig. 1b bottom, Supplementary Fig. S3), indicating that MreB5 is involved in both helix formation and movements.

Next, to examine the subcellular localization of MreB4, we labeled it by inserting mCherry immediately after Y225, a position corresponding to the labeling site of MreB5. Signals were observed in almost all the cells, and some cells showed helical shape. However, no moving cells were observed (Fig. 1c, Supplementary Fig. S4). Then, we moved the insertion positions by one residue; however, none of the 14 constructs showed cell movements (Supplementary Fig. S4). The difficulty in fluorescence labeling of MreB4 may suggest that MreB4 interacts with other structures through larger numbers of positions than MreB5. Then we tried to label MreB4 by small tetracysteine (TC) motif composed of only 12 amino acid residues (FLNCCPGCCMEP). Biarsenical dye called FlAsH is expected to conjugate this motif and emit fluorescence [24,25]. We fused TC motif at N-terminus, C-terminus, and immediately after Y225 of MreB4. The construct inserted immediately after Y225 showed 5 cells moving out of approximately 500 (Supplementary Movie S3). However, helicity and kink were not observed even they showed repeated winding motion due to the short cell length. Moreover, the background fluorescence probably from nonspecific binding of FlAsH was high. Then, we concluded that the TC labeling was not suitable for analysis of MreB cellular dynamics.

### Relation between expression level of each MreB and motility

**Figure 2:**
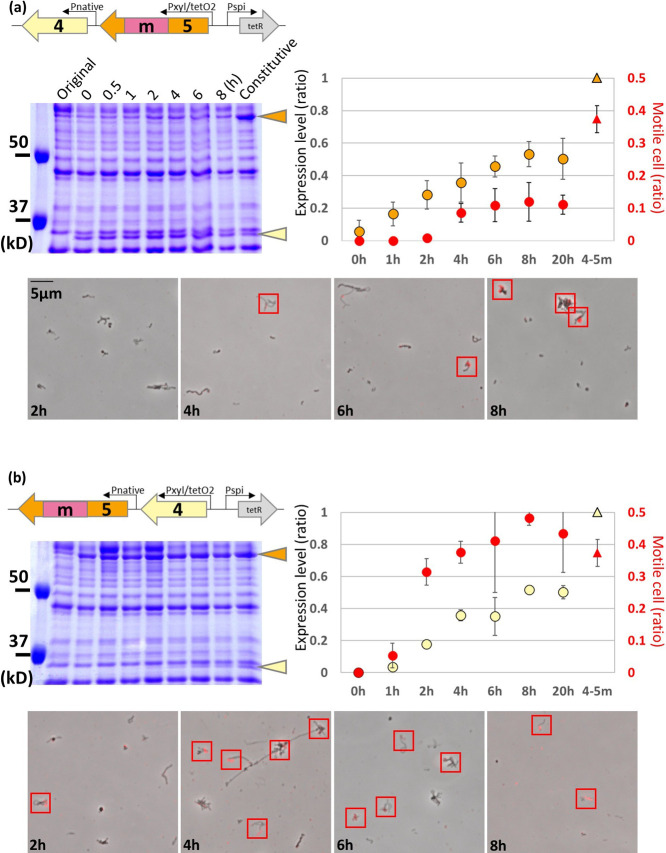
Motile cell ratio at various times after one MreB induction in a background of constitutive expression of another MreB. (a) MreB5 induction to constitutive MreB4 expression. (b) MreB4 induction to constitutive MreB5 expression. (a, b) Gene constructs are illustrated in the upper left. Protein profiles at various induction times were analyzed by SDS-10%-PAGE. The bands for MreB4 and MreB5m are marked by yellow and orange triangles, respectively. Cell images at various induction times are presented on the bottom. The moving parts are colored red. Motile cells are marked by a red square. The integrated results (n=3) are shown in the upper right. Red circles show the ratio of moving cells. Yellow and orange circles respectively show the band intensities of MreB4 and MreB5m normalized by constitutively expressed MreB4 and MreB5m in the strain pSeN540mC5 (4-5m) that are shown by triangles.

We examined the ratio of motile cells at various expression levels of each MreB. We constructed the strain with a constitutive expression of an MreB and another inducible one. In these strains, one MreB is under the control of the Pxyl/tetO2 promoter, where the expression is prevented by a tetracycline repressor (TetR) [26]. The MreB expression was induced by adding 9 μM tetracycline. The protein levels and ratio of motile cells were then analyzed (Fig. 2ab, Supplementary Movie S4). In the strain expressing both MreBs constitutively, the ratio of moving cells was 37.4±4.2%. In the strain expressing only MreB5m constitutively, motile cell ratio reached 37.6±3.4% when MreB4 was induced to 35.7±3.4% of the constitutive level. On the other hand, in the strain expressing only MreB4 constitutively, MreB5m induction to the 35.7±1.2% resulted in 8.6±2.9% motile cells. Higher requirement for MreB5 than MreB4 to move the cells is consistent with previous suggestions that MreB5 serves as the backbone of the helix and that MreB4 causes MreB5 helix inversion [14,27,28]. This may be related to the fact that MreB1, which is thought to function similarly as MreB4, has high ATPase activity [27]. However, it should be noted that the expression level shown here is for the whole culture and not for individual cells.

### MreB5 replacement traced by photobleaching and photoactivation

**Figure 3:**
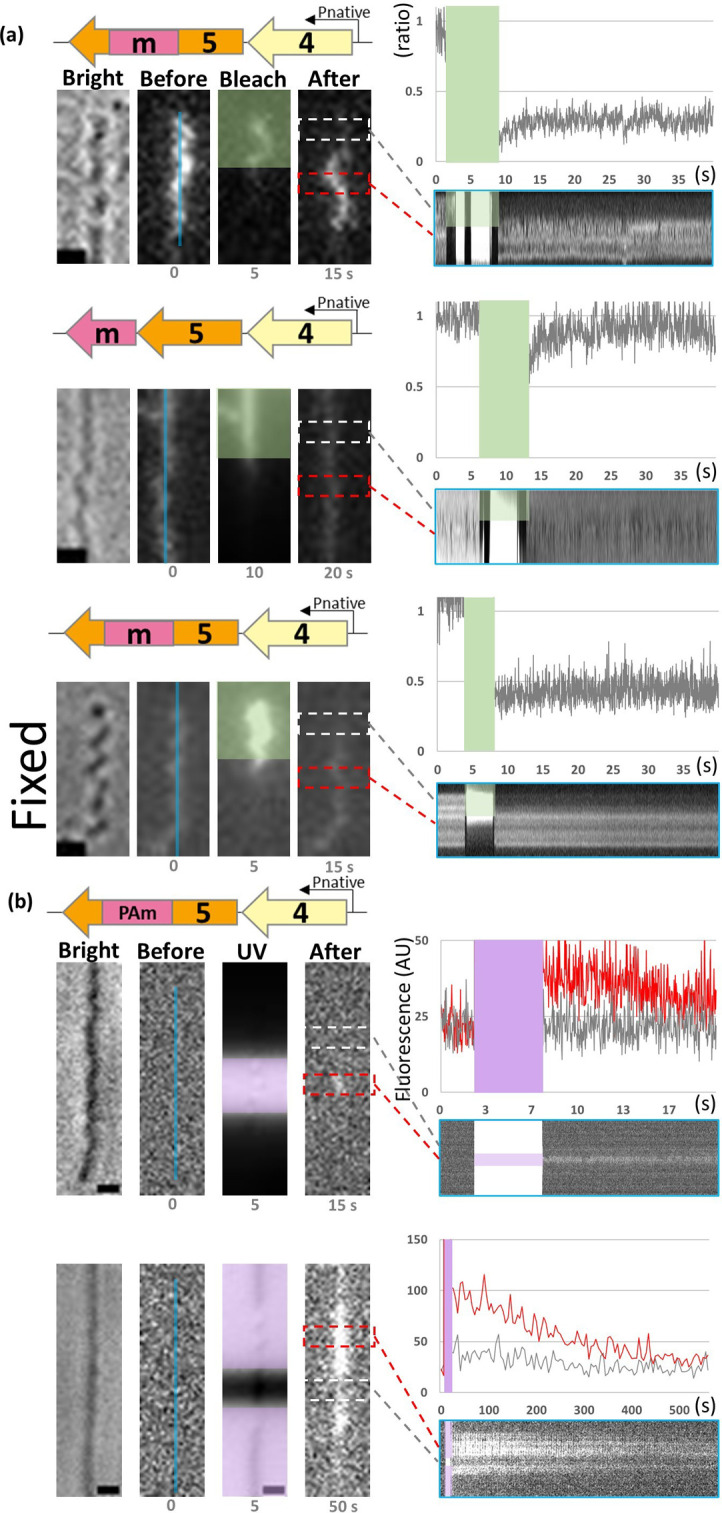
MreB5 dynamics examined by photobleaching and photoactivation. In each block, gene constructs are illustrated in the left upper. Cell images are shown in the left lower. Scale bar, 1 μm. Fluorescence traces are shown in the right upper. Kymographs along the blue lines are shown in the right lower. Timeline is common to fluorescence trace and kymograph. (a) Photobleaching. Cell images in brightfield, prebleach, bleach, and postbleach are shown. Bleached area is shown by a green translucent rectangle. Recovery of bleached fluorescence was traced for 40 s by measuring the fluorescence intensity ratio of white to red boxed areas. Bleaching time is shown by a green rectangle. (b) Photoactivation. Cell images in brightfield, preactivation, UV irradiation, and postactivation are shown. UV irradiated area is shown by a purple translucent rectangle. Fluorescence intensity for red and white boxed areas are traced. UV irradiation time is shown by a purple rectangle.

MreB generally has polymerization and ATPase activities like eukaryotic actin [16]. In *Spiroplasma*, MreB5 polymerizes into filaments in the presence of ATP [29]. To examine whether frequent polymerization-depolymerization is involved in the helicity switching, we focused on MreB5m behaviors in a moving cell. The FRAP is a technique to measure exchange dynamics of target proteins from the recovery rate of the fluorescence intensity at the photobleached area [30]. If MreB5 subunit replacement is frequent, fluorescence should recover rapidly. Here, we focused on cells that were attached to glass only at poles and stretched by liquid flow applied before observation. These cells exhibited movements, although the kink propagation did not fully reach the pole due to the attachment. We bleached one end of such cells and traced the fluorescence intensities as shown by a kymograph (Fig. 3a). In the strain expressing MreB4 and MreB5m (Fig. 3a upper, Supplementary Movie S5), fluorescence intensity dropped to about 10% of initial value and returned to a plateau at about 30% within 5 s. In the strain expressing free mCherry additionally to MreB4 and MreB5 (Fig. 3a middle), the post-bleach intensity reached over 95% of initial value within 10 s. In chemically fixed cells (Fig. 3a bottom), the recovery was not observed. The latter two results proved that free fluorescent molecules spread quickly but fixed ones cannot. The recovery to 30% in the strain expressing MreB4 and MreB5m indicates the presence of diffusive MreB5m. However, the lack of subsequent recovery suggests that the diffusive molecules are not incorporated into MreB5m filaments. Then, to focus on the behaviors of MreB forming filaments, we tried photoactivation using PAmCherry (Photoactivatable mCherry) [31]. This protein is initially non-fluorescent and activated by UV irradiation. The photoactivation is achieved by only 10 amino acid modification from mCherry. The MreB5 fused with PAmCherry retained functions for cell elongation and movements, as observed for mCherry fusion. Then, we irradiated the cells with UV light, and traced the subsequent fluorescence intensity (Fig. 3b, Supplementary Movie S6). After UV irradiation, only the irradiated area emitted fluorescence. The fluorescence intensity decreased gradually due to the photobleaching; however, there was no obvious inflow or outflow of MreB5m between bright and dark regions. This is consistent with the result of FRAP (Fig. 3a).

These results indicate that MreB5 remains in the same cellular position during movements, without obvious replacements, which seems to be distinct from other bacterial actins such as ParM and MamK that exhibit dynamic polymerization [32,33].

### Effect of MreB polymerization inhibitor A22 on cell movements and MreB5 localization

**Figure 4:**
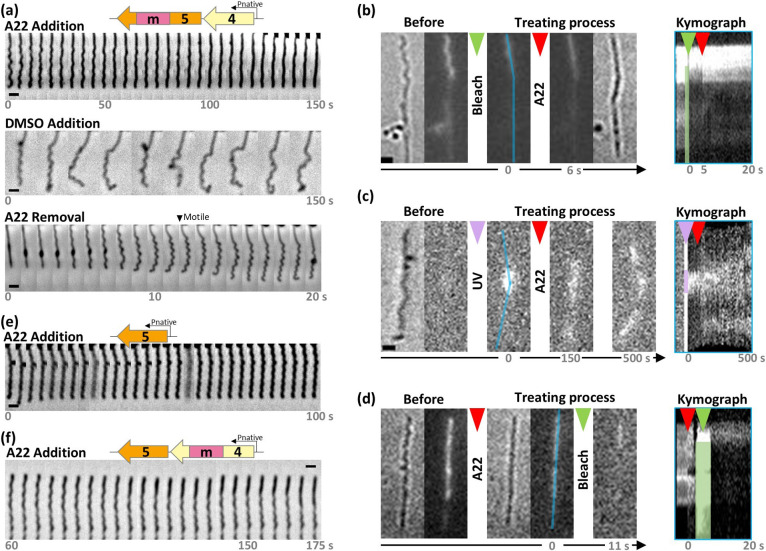
Effects of A22 on cell movements, shape, and MreB5m behaviors. (a) Change in cell shape after addition (upper) and removal (bottom) of A22. DMSO was used for control (middle). Gene construct is illustrated on the top. (b)-(d) Effects of A22 addition on MreB5 behaviors. Cell images in the process are shown in order. Kymograph shows the fluorescence distribution along the cell axis, which is marked by a blue line. The time count on the bottom of each image starts after the photobleaching, photoactivation, and A22 addition. (b) Cells were first photobleached (green triangle and translucent thin rectangle) and treated with A22 (red triangle). Kymograph does not include the bleaching image. (c) Cells were first photoactivated (purple triangle and translucent thin rectangle) and treated with A22 (red triangle). For kymograph, the blue line for the cell axis was slightly adjusted with time. (d) Cells were first treated with A22 (red triangle) and photobleached (green triangle and translucent rectangle). (e) A22 addition to the cell expressing only MreB5. (f) A22 addition to the cell expressing MreB5 and labeled MreB4.

To know the role of the polymerization ability of MreB, we examined the effects of an MreB polymerization inhibitor A22. A22 is a compound with a molecular weight of 271.6 and has been shown to bind the nucleotide binding site of *Caulobacter crescentus* MreB [18,34]. Addition of 250 μM A22 to the syn3B cells expressing MreB4 and MreB5m linearized the helix, and stalled movements in 150 s (Fig. 4a-upper, Supplementary Movie S7). To examine if the intracellular MreB5 changes to diffusive, we added A22 after the photobleaching in FRAP observation. Fluorescence once reached a plateau after bleaching and recovered to 20% of original, after A22 addition (n=2) (Fig. 4b). To observe this change more clearly, we added A22 to the cell expressing MreB4 and MreB5-PAmCherry after photoactivation by the UV irradiation. Fluorescence of the specific photoactivated region was observed to be spread in 100 s after A22 addition (Fig. 4c). These results indicate that A22 inhibited some interaction responsible for MreB positioning in a cell. Next, we initially linearized the cell by A22, then performed FRAP observation. If the MreB5 is released from all interactions, fluorescence trace is expected to be like free mCherry (Fig. 3a middle). However, no fluorescence recovery was observed (Fig. 4d). This result may suggest that some MreB5m molecules stay at their positions even after the cell linearization. This may be consistent with the uneven PAmCherry fluorescence distribution after A22 addition (Fig. 4c).

Besides the above experiments, we observed the cell changes after the A22 removal to know if this motility inhibition is reversible or not. A22 removal caused helix reformation immediately and the motility recovered within 10 s (Fig. 4a-bottom, Supplementary Movie S8). Since both helix linearization and reformation occurred along the entire cell axis, MreB5 was unlikely released only at cell poles.

We added A22 to the immotile cells. Cells expressing intact MreB5 without MreB4 were hardly linearized by A22 (Fig. 4e), Supplementary Movie S9). We also added A22 to a cell with intact MreB5 and labeled MreB4 (Fig. 4f). As a result, only 40% was linearized after 110–175 s from A22 addition (Fig. 4a). This observation can be explained by two possibilities. One is that the A22 binding site of MreB5 is not accessible in the absence of movements. Another possibility is that A22 binds to MreB protein, but the conformational change does not occur.

## Conclusion

**Figure 5:**
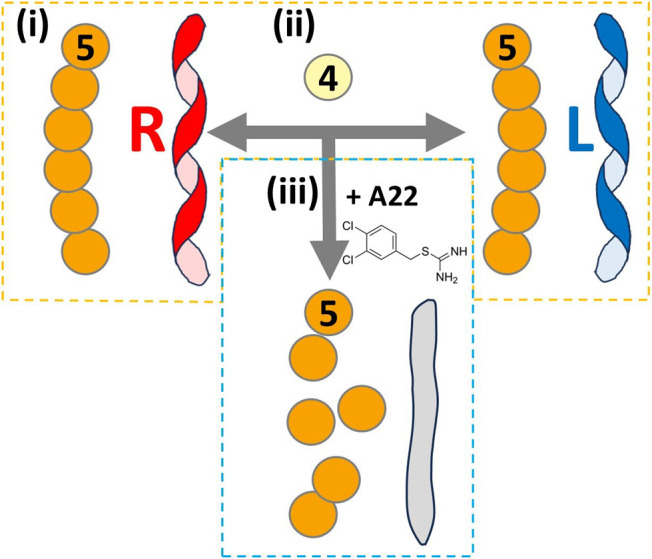
Summary of this study. Suggestion numbers (i, ii, iii) are placed at corresponding positions. MreB4 switches right-handed cell to left-handed with no obvious replacement of MreB5 molecules responsible for the cell helicity (Orange dashed line). Addition of polymerization inhibitor A22 causes helix deformation, movement stall, and diffusion of MreB5 molecules when the cell is moving (Blue dashed line).

This study gives following suggestions (Fig. 5), which are consistent with previous studies, as follows. (i) MreB5 forms filamentous helical structures (Fig. 2)[28,35–37]. (ii) Helicity switching of MreB5 filament is caused by MreB4 (Figs. 1 and 2)[14,27] without a dynamic replacement (Fig. 3), (iii) Filament formation of MreB5 can be affected by A22 (Fig. 4).

## Conflict of interest

We declare no conflicts of interest.

## Author contributions

YT performed most of the experiments and wrote the original draft. HK performed experiments described in the section “New constructs for adequate protein expression”, and provided the strategy of inducible MreB constructs. YVM provided the photoactivation experiment with equipment. YT and MM edited the manuscript. All authors contributed to the project and manuscript.

## Data availability

The evidence data generated/analyzed in this study are included in this article.

A preliminary version of this work, DOI: https://doi.org/10.1101/2025.05.23.655722, was deposited in the bioRxiv on May 25, 2025.
