# Supplementary material for: Live imaging of bacterial actin MreBs from Spiroplasma causing helicity switching of a minimal synthetic cell
Source: Biophys Physicobiol. 2026 May 13;23(2):e230017. doi: 10.2142/biophysico.bppb-v23.0017 (PMC13310577; doi:10.2142/biophysico.bppb-v23.0017)
Supplement: Supplementary file 1 — Supplementary Materials [file 23_e230017_1.pdf]

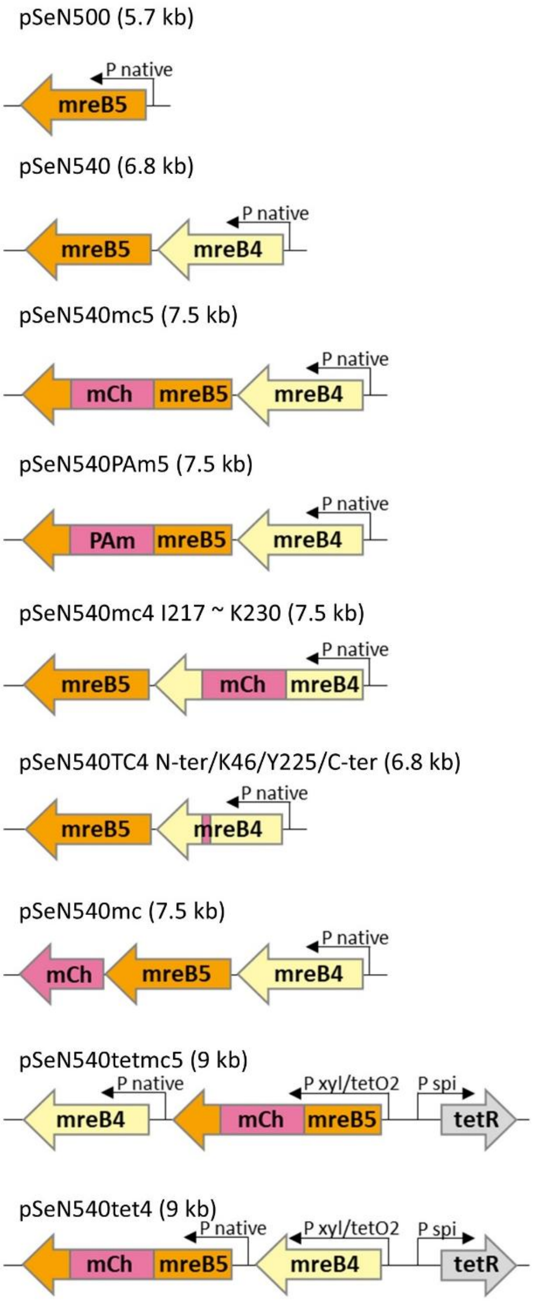

Supplementary Figure S1 Schematic presentation for DNA constructs used in this study.

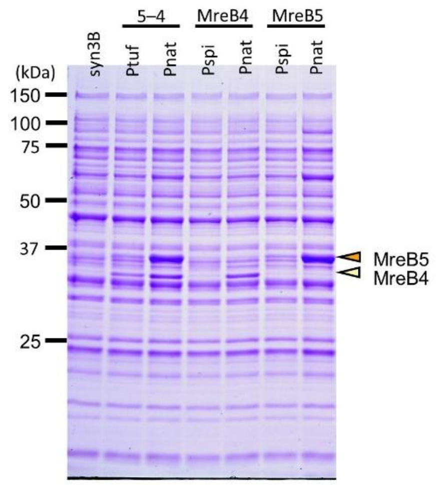

Supplementary Figure S2 Comparison of expression level of MreB4 and MreB5 between previous and improved promoters, by SDS-12.5% PAGE. The bands for MreB4 and MreB5 are marked by yellow and orange triangles, respectively.

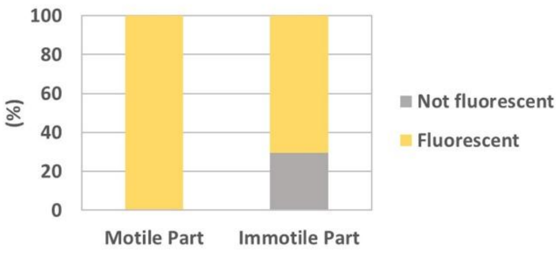

Supplementary Figure S3 The ratio of fluorescent parts at moving and non-moving parts of cells harboring pSeN540mc5.

(a)

Position	FP, Tag	Helix	Movement
N-ter	EYFP	×	×
	TC	○	×
K46-E47	EYFP	○	×
	TC	○	×
E218-I219	mCherry	○	×
I219-G220	mCherry	○	×
G220-S221	mCherry	△	×
S221-L222	mCherry	○	×
L222-A223	mCherry	○	×
A223-K224	mCherry	×	×
K224-Y225	mCherry	△	×
Y225-P226	mCherry	○	×
	TC	○	△
P226-D227	mCherry	○	×
D227-E228	mCherry	○	×
E228-R229	mCherry	○	×
R229-K230	mCherry	○	×
K230-M231	mCherry	○	×
C-ter	EYFP	○	×
	TC	△	×

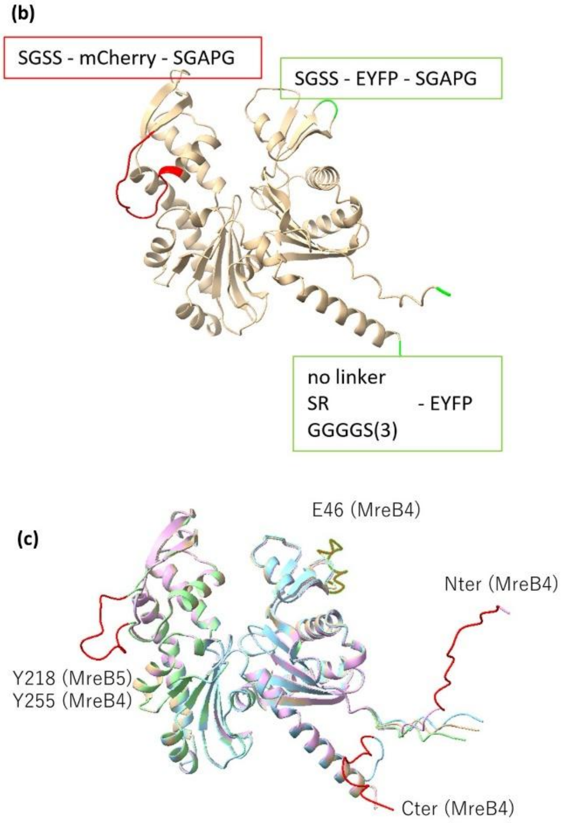

Supplementary Figure S4 Characterizing transformants of MreB4 fused with mCherry, EYFP, and TC-tag. (a) Morphology and movements. (b) Labeled positions indicated in MreB4 structure predicted by AlphaFold2. (c) Predicted structure of MreB4 labeled with TC-Tag.

Supplementary Table S1 DNA primers used in this study.

Name	Sequence	Purpose	Template	Assembly
Lp_inverse_F	AGGACTGAGCTAGCTGTCAAAGATC	pSeN540	pSD079	iVEC3
LP-inverse-R	GAACTATATAAATAACTCGCATATTG	pSeN540	pSD079	iVEC3
		pSeN400	pSeN540	iVEC3
		4-tet5m, 4-tet5	pSD079	In Fusion
opr-mreB4-5-079-F	AATATGCGAGTTATTTATATAGTTCATTTGGCATCCAAGAATAACG	pSeN540	*S. eriocheiris* genome	iVEC3
opr-mreB4-5-079-R	GATCTTTGACAGCTAGCTCAGTCCTCTATTGCTAATTTCAAGAAGTCAT	pSeN540	*S. eriocheiris* genome	iVEC3
N540_delMreB4_F	AAATATATTCTGTAAAAATTCCTCCCTTT	pSeN500	pSeN540	iVEC3
N540_delMreB4_R	AATTTTTACAGAATATATTTAGAGAAATTCGCTT	pSeN500	pSeN540	iVEC3
MreB4-F	GCGAGTTATTTATATAGTTCTTAGTAATCTAATTCTTTAGTATG	pSeN400	pSeN540	iVEC3
mreB5-mChe-link-F	ATAATAGCTGATGATCCTGAGTATTTAACTAATGAACCGATGTA	pSeN540mc5	pSeN540	In Fusion
mreB5-mChe-link-R	ATAAAAGTGGAGCTCCTGGTCATAATGAACGTGCAATGCAAATT	pSeN540mc5	pSeN540	In Fusion
SWmChe-linker-F	ACCAGGAGCTCCACTTTTATATAGTTCATCCATACCAC	pSeN540mc5	pSeW545-F5	In Fusion
SWmChe_linker_R2	TCAGGATCATCAGCTATTATTAAAGAATTT	pSeN540mc5	pSeW545-F5	In Fusion
del-dCas9-F	CTTGTTGCCTCCTTAGCAGATGCT	4-tet5m, 4-tet5	pSD079	In Fusion
Vector_MreB4_TY_F	GCGAGTTATTTATATAGTTCTTAGTAATCTAATTCTTTAGTATGC	4-tet5m, 4-tet5	pSeN540	In Fusion
MreB4_MreB5_TY_R	GATGCCAAATCTATTGCTAATTTCAAGAAGTCATT	4-tet5m, 4-tet5	pSeN540mc5	In Fusion
MreB4_MreB5_TY_F	TTAGCAATAGATTTGGCATCCAAGAATAACGGATT	4-tet5m, 4-tet5	pSeN540mc5	In Fusion
MreB5_vector_TY_R	TCTGCTAAGGAGGCAACAAGGTGAAACCAGAAAGACCATTTATCT	4-tet5m, 4-tet5	pSeN540	In Fusion
N540_delMreB4_F	AAATATATTCTGTAAAAATTCCTCCCTTT	5-tet4	4tet5m	NEB
TetMreB4_VR	AGATTACTAAGCTGGCATTGGGAAGAAA	5-tet4	4tet5m	NEB
TetMreB4_IF	CAATGCCAGCTTAGTAATCTAATTCTTTAGTATGCATTTGTTC	5-tet4	pSeN540	NEB
TetMreB4_IR	TCTGCTAAGGAGGCAACAAGATGGCAGGATTTAATAGC	5-tet4	pSeN540	NEB
del-dCas9-F	CTTGTTGCCTCCTTAGCAGATGCT	5-tet4	45tet5H	NEB
N540_delMreB4_R	AATTTTTACAGAATATATTTAGAGAAATTCGCTT	5-tet4	45tet5H	NEB
m_PAm_IF	TATCTAATTTTCTATTAACATTATAAGC	4-5PAmCherry	PAm-fragment	NEB
m_PAm_IR	TAATGGACATGTTTTTGAAATAGAAGGTG	4-5PAmCherry	PAm-fragment	NEB
PAm_m_VF	TTTCAAAAACATGTCCATTAACTGATCC	4-5PAmCherry	pSeN540mc5	NEB
PAm_m_VR	TGTTAATAGAAAATTAGATATTACTTCAC	4-5PAmCherry	pSeN540mc5	NEB
MreB5ter-mCherry-IF	TCTTGTTAATTATTTATATAGTTCATCCATACCAC	4-5-mCherry	pSeN540mc5	In Fusion
MreB5-MreB5SD-mCherry-IR	TATTAAGGAGGAAATTAACATGGCTATTATTAAAGAATT	4-5-mCherry	pSeN540mc5	In Fusion
mCherry-MreB5SD-MreB5-VF	TGTTAATTTCCTCCTTAATATTTATTTTTTCTTCCCAATG	4-5-mCherry	pSeN540	In Fusion
mCherry-MreB5ter-VR	GGATGAACTATATAAATAATTAACAAGATAAATTAAA	4-5-mCherry	pSeN540	In Fusion
syn3B-junc-F	TATGTGATAATGCCAATCGCTAAG	colonyPCR		
syn3B-junc-R	GTAAATTCCCAAATTATTCCATCA	colonyPCR		
MreB4_TC_Nter_F	TACAACAACCTGGACAACAATTTAAAAACATTGTAAAAATTCCTCC	4TC(Nter)-5	pSeN540	iVEC3
MreB4_TC_Nter_R	TTGTTGTCCAGGTTGTTGTATGGAACCAGCAGGATTTAATAGCGGC	4TC(Nter)-5	pSeN540	iVEC3
MreB4_TC_Cter_F	TACAACAACCTGGACAACAATTTAAAAAGTAATCTAATTCTTTAG	4TC(Cter)-5	pSeN540	iVEC3
MreB4_TC_Cter_R	TTGTTGTCCAGGTTGTTGTATGGAACCATAAAAAAGAATATATTTAG	4TC(Cter)-5	pSeN540	iVEC3
MreB4_TC_Y225P226_F	CAACAACCTGGACAACAATTTAAAAAGTATTTTGCTAATGAACC	4TC(Y225)-5	pSeN540	iVEC3
MreB4_TC_Y225P226_R	AATTGTTGTCCAGGTTGTTGTATGGAACCAGACGAAAGAAAAATG	4TC(Y225)-5	pSeN540	iVEC3
MreB4_TC_46K47E_F	TACAACAACCTGGACAACAATTTAAAAATTTTATTCTGTAAGCTAC	4TC(K46)-5	pSeN540	iVEC3
MreB4_TC_46K47E_R	TTGTTGTCCAGGTTGTTGTATGGAACCAGAAAATAGAATTATTGC	4TC(K46)-5	pSeN540	iVEC3
MreB5_TC_Y218H219_F	TACAACAACCTGGACAACAATTTAAAAAGTATTTAACTAATGAACC	4-5TC(Y218)	pSeN540	iVEC3
MreB5_TC_Y218H219_R	TTGTTGTCCAGGTTGTTGTATGGAACCACATAATGAACGTGCAATG	4-5TC(Y218)	pSeN540	iVEC3
SWmChe_linker_F2	ACCAGGAGCTCCACTTTTATATAG	4m-5	pSeN540mc5	In Fusion
SWmChe_linker_R2	TCAGGATCATCAGCTATTATTAAAGAATTT	4m-5	pSeN540mc5	In Fusion
pSeW217_F	ATAATAGCTGATGATCCTGAAATTTTAATTTGTTCAGC	4m-5	pSeN540	In Fusion
pSeW217_R	ATAAAAGTGGAGCTCCTGGTGAAATTGGTTCATTAGCAAA	4m-5	pSeN540	In Fusion
pSeW218_F	ATAATAGCTGATGATCCTGATTCAATTTTAATTTGTTCAG	4m-5	pSeN540	In Fusion
pSeW218_R	ATAAAAGTGGAGCTCCTGGTATTGGTTCATTAGCAAAATA	4m-5	pSeN540	In Fusion
pSeW219_F	ATAATAGCTGATGATCCTGAAATTTCAATTTTAATTTGTTC	4m-5	pSeN540	In Fusion
pSeW219_R	ATAAAAGTGGAGCTCCTGGTGGTTCATTAGCAAAATAC	4m-5	pSeN540	In Fusion
pSeW220_F	ATAATAGCTGATGATCCTGAACCAATTTCAATTTTAATTTG	4m-5	pSeN540	In Fusion
pSeW220_R	ATAAAAGTGGAGCTCCTGGTTCATTAGCAAAATACCCAG	4m-5	pSeN540	In Fusion
pSeW221_F	ATAATAGCTGATGATCCTGATGAACCAATTTCAATTTTAATTTG	4m-5	pSeN540	In Fusion
pSeW221_R	ATAAAAGTGGAGCTCCTGGTTTAGCAAAATACCCAGAC	4m-5	pSeN540	In Fusion
pSeW222_F	ATAATAGCTGATGATCCTGATAATGAACCAATTTCAATTTTAATTTG	4m-5	pSeN540	In Fusion
pSeW222_R	ATAAAAGTGGAGCTCCTGGTGCAAAATACCCAGACGAAAG	4m-5	pSeN540	In Fusion
pSeW223_F	ATAATAGCTGATGATCCTGATGCTAATGAACCAATTTCAA	4m-5	pSeN540	In Fusion
pSeW223_R	ATAAAAGTGGAGCTCCTGGTAAATACCCAGACGAAAGAAA	4m-5	pSeN540	In Fusion
pSeW224_F	ATAATAGCTGATGATCCTGATTTTGCTAATGAACCAATTTC	4m-5	pSeN540	In Fusion
pSeW224_R	ATAAAAGTGGAGCTCCTGGTTACCCAGACGAAAGAAAA	4m-5	pSeN540	In Fusion
pSeW225_F	ATAATAGCTGATGATCCTGAGTATTTTGCTAATGAACCAA	4m-5	pSeN540	In Fusion
pSeW225_R	ATAAAAGTGGAGCTCCTGGTCCAGACGAAAGAAAAATGAA	4m-5	pSeN540	In Fusion
pSeW226_F	ATAATAGCTGATGATCCTGATGGGTATTTTGCTAATGAAC	4m-5	pSeN540	In Fusion
pSeW226_R	ATAAAAGTGGAGCTCCTGGTGACGAAAGAAAAATGAAAG	4m-5	pSeN540	In Fusion
pSeW227_F	ATAATAGCTGATGATCCTGAGTCTGGGTATTTTGCTAATG	4m-5	pSeN540	In Fusion
pSeW227_R	ATAAAAGTGGAGCTCCTGGTGAAAGAAAAATGAAAGTTTA	4m-5	pSeN540	In Fusion
pSeW228_F	ATAATAGCTGATGATCCTGATTCGTCTGGGTATTTTGC	4m-5	pSeN540	In Fusion
pSeW228_R	ATAAAAGTGGAGCTCCTGGTAGAAAAATGAAAGTTTATGG	4m-5	pSeN540	In Fusion
pSeW229_F	ATAATAGCTGATGATCCTGATCTTTCGTCTGGGTATTTTG	4m-5	pSeN540	In Fusion
pSeW229_R	ATAAAAGTGGAGCTCCTGGTAAAATGAAAGTTTATGGACG	4m-5	pSeN540	In Fusion
pSeW230_F	ATAATAGCTGATGATCCTGATTTTCTTTCGTCTGGGTATT	4m-5	pSeN540	In Fusion
pSeW230_R	ATAAAAGTGGAGCTCCTGGTATGAAAGTTTATGGACGTG	4m-5	pSeN540	In Fusion

Movie S1: Field images of syn3B cells expressing MreBs by three different promoters.

Movie S2: Moving syn3B cell expressing MreB4 and MreB5.

Movie S3: Field images of syn3B cells expressing MreB5 fused with TC at different positions.

Movie S4: Field images of syn3B cells after induction of MreB5 and MreB4, under constitutive expression of MreB4 and MreB5, respectively.

Movie S5: Movie of Figure 3a-upper.

Movie S6: Movie of Figure 3b-upper.

Movie S7: Movie of Figure 4a-upper.

Movie S8: Movie of Figure 4a-bottom.

Movie S9: Movie of Figure 4e.
